# A Compact High-Efficiency Tri-Band CPW-Fed Millimeter-Wave Dielectric Patch Antenna for Wireless Systems

**DOI:** 10.3390/s26185788

**Published:** 2026-09-11

**Authors:** Ali-Abdullah Albarakati, Salam K. Khamas

**Affiliations:** 1 Department of Electrical Engineering, Umm Al-Qura University, Makkah 24382, Saudi Arabia; 2 Department of Electronic and Electrical Engineering, University of Sheffield, Sheffield S10 2TN, UK; s.khamas@sheffield.ac.uk

**Keywords:** 5G, coplanar waveguide, dielectric patch antenna, millimetre-wave antennas, multiband antennas

## Abstract

A low-profile tri-band millimeter-wave (mmWave) dielectric patch (DP) antenna (DPA) is proposed for fifth-generation (5G) and beyond wireless communication systems. Multiband operation is achieved by exciting the resonant ${\mathrm{TM}}_{11}$, ${\mathrm{TM}}_{31}$, and ${\mathrm{TM}}_{51}$ modes at approximately 22, 30, and 36 GHz, respectively. Crucially, these operating bands are strategically designed to align with essential mmWave communication standards: the 22 GHz band targets fixed short-range microwave backhaul links; the 30 GHz band services 5G New Radio (NR) n257/n258 allocations; and the 36 GHz band accommodates early 5G NR n260 spectrum requirements alongside emerging radar and sensing applications. The three modes are excited using a coplanar waveguide (CPW) feed structure. A prototype was fabricated and experimentally characterized, and its measured reflection coefficient, realized gain, and radiation patterns were compared with full-wave simulations. The proposed DPA exhibits an overall profile of $0.086\lambda_{0}$ while providing impedance bandwidths of 4.57% (21.4–22.4 GHz), 4.24% (30–31.3 GHz), and 2.75% (35.8–36.8 GHz), together with simulated radiation efficiencies of 93%, 95%, and 95% across the three respective bands. In addition, it delivers measured peak realized gains of 8.3, 5.2, and 4.3 dBi at 22, 30, and 36 GHz, respectively. The proposed antenna combines tri-band coverage, a low profile, and high simulated efficiency in a single compact radiating element, making it a highly compelling candidate for modern compact multiband mmWave wireless system infrastructures.

## 1. Introduction

The deployment of 5G New Radio (NR) and emerging 6G architectures is driving wireless systems toward mmWave frequencies to enable multi-gigabit data rates, ultra-low latency, and massive connectivity for enhanced mobile broadband (eMBB), vehicle-to-everything (V2X), and Internet-of-Things (IoT) applications [1,2,3]. These applications demand compact, multiband RF front ends that support diverse mmWave standards while operating within stringent space constraints. Consequently, a single-aperture, multiband antenna solution is essential to ensure seamless operation across heterogeneous networks while maintaining a low profile and fabrication simplicity. The three targeted operating frequencies correspond to key mmWave wireless standards: the 22 GHz band is widely utilized in fixed short-range backhaul links; the 30 GHz band closely aligns with the 5G NR n257/n258 spectrum allocations; and the 36 GHz band aligns with the 5G NR n260 spectrum and emerging radar and sensing applications.

To meet this demand, various planar and three-dimensional antenna configurations have been explored. Conventional microstrip patch antennas (MPs) have been extensively investigated for multiband mmWave operation [4,5,6,7,8,9,10,11,12,13]. Nevertheless, they can suffer from narrow impedance bandwidth and increased conductor losses at mmWave frequencies, which may reduce radiation efficiency, particularly in compact or multilayer implementations. For instance, the tri-band MP designs reported in [9,13] exhibit efficiencies as low as 30% and 54%, respectively, while several other MP-based multiband designs [4,5] do not report efficiency data, preventing assessment of their radiation performance. Although certain MP configurations achieve moderate efficiencies in the range of 79–95% [6,7,10], they either lack a low-profile architecture or rely on complex multilayer stacking with profiles reaching up to 0.48λ [10].

Dielectric resonator antennas (DRAs) have also been explored for dual-band and multiband mmWave operation, offering inherent advantages such as low ohmic loss, high radiation efficiency, and greater geometric design freedom compared with their microstrip counterparts. For example, a compact dual-band two-element rectangular DRA MIMO array in which each element is excited by a coplanar waveguide (CPW) feed through a dedicated ground-plane slot is proposed in [14]. The two 3D-printed alumina (with $\varepsilon_{r}=9.9$) resonators are mounted on opposite sides of a Rogers RO4003 substrate and each antenna resonates at the ${\mathrm{TE}}_{111}$ and ${\mathrm{TE}}_{311}$ modes, yielding −10 dB impedance bandwidths of 18% and 13% centered at 28 GHz and 38 GHz, respectively. In addition, ref. [15] demonstrated a dual-band circularly polarized (CP) rectangular DRA in which a modified cross-flower (MCF) slot, etched in the ground plane of a Rogers RT/duroid 5880 substrate and excited by a microstrip line terminated in a hexagonal pad, couples simultaneously to the fundamental (${\mathrm{TE}}_{\delta 11}^{x}/{\mathrm{TE}}_{1\delta 1}^{y}$) and higher-order (${\mathrm{TE}}_{\delta 13}^{x}/{\mathrm{TE}}_{1\delta 3}^{y}$) modes of a magnesium-metasilicate ceramic resonator ($\varepsilon_{r}=7$, tanδ=0.005). The hybrid slot–DRA structure achieves −10 dB impedance bandwidths of 23.3% (23.9–30.2 GHz) and 25% (35.3–45.4 GHz), together with 3 dB axial-ratio bandwidths of 19.8% and 7.8% and peak gains of 5.62 dBic and 6.74 dBic in the lower and upper bands, respectively. Furthermore, ref. [16] reported a multiband mmWave rectangular DRA fed by a planar rectangular ring-slot etched in a Rogers RO4003 ground plane. By exciting the fundamental ${\mathrm{TE}}_{111}$ mode at 17.5 GHz, the ring-slot resonance at 23 GHz, and the degenerate higher-order ${\mathrm{TE}}_{121}^{x}/{\mathrm{TE}}_{211}^{y}$ modes at 28.5 GHz, the alumina ($\varepsilon_{r}=9.9$) resonator achieves triple-band operation with measured bandwidths of 3.4%, 7.7% and 1.9% and realized gains of 6.56 dBi, 5.2 dBi and 4.33 dBi, respectively, while maintaining a stable omnidirectional pattern and a radiation efficiency of approximately 90% across all bands. Collectively, these studies demonstrate that dual- and multiband DRAs can provide efficient, compact and independently controllable multiband performance for mmWave 5G and beyond-5G systems. However, they typically rely on modified DR geometries with $\varepsilon_{r}\approx 10$, which motivates the present work to use a rectangular dielectric patch (DP) resonator with a high permittivity of ($\varepsilon_{r}=82$) to achieve multiband with three resonances across the 20–40 GHz range.

Dielectric patch (DP) antennas (DPAs) have emerged as promising candidates for modern wireless systems owing to their low profile, high radiation efficiency, and compatibility with planar integration [17,18]. To position the present work within the broader literature, this section reviews representative DPA research organized along four principal areas that have shaped recent progress in the field, namely bandwidth enhancement [19,20,21,22], filtering response [23,24,25,26,27], multi-element MIMO configurations [28,29,30,31,32], and circular polarization [33,34,35,36].

Existing research has explored bandwidth enhancement in DPAs by intentionally merging multiple resonant modes. Ref. [19] introduced multiple air regions within the dielectric resonator to independently manipulate the resonant frequencies of the $TM_{101}$, $TM_{121}$, $TM_{321}$, and $TM_{141}$ modes, achieving a measured bandwidth of 44.9% (8.3–13.1 GHz) with stable radiation patterns across the X-band. Ref. [20] etched three pairs of differently sized slots into a monolithic dielectric patch to merge the $TM_{10}$ and $TE_{12}$ modes while simultaneously improving the isolation between TX and RX ports for in-band full-duplex operation, attaining 18% bandwidth with isolation greater than 28 dB. Ref. [21] employed four metallic pins inside a dielectric patch resonator to shift the $TM_{121}$ mode downward and the $TM_{101}$ mode upward, bringing the two modes into close proximity and yielding a dual-polarized response with 15.2% bandwidth at a low profile of 0.07λ_0_. Ref. [22] exploited a dual-slot-coupled multi-patch configuration to excite five modes simultaneously, two from the dielectric patches and three from the slots, achieving a wide impedance bandwidth of 81.9% around 8.98 GHz with a peak gain of 9.27 dBi. Collectively, these works demonstrate that mode manipulation, whether through air regions, slot etching, metallic pins, or hybrid patch–slot structures, is the principal route to bandwidth enhancement in DPA design.

Filtering response in DPAs has been realized by merging multiple modes inside the DP and by introducing features that generate paired radiation nulls at the passband edges, eliminating the need for external filter circuitry. Ref. [23] excited the $TE_{12}$, $TE_{12+\delta }$, and $TE_{13}$ modes of a DP through a four-slot feed and a U-shaped microstrip line, generating a lower-edge null from a parallel four-slot ground array and an upper-edge null from a non-radiative mode of the dielectric, with rectangular grooves, an L-shaped defect, and a C-shaped stub used to refine matching and stopband suppression, achieving a 13.2% bandwidth (4.6–5.25 GHz) and a peak realized gain of 9.85 dBi at a profile of $0.069\lambda_{0}$. Ref. [24] fed the $TM_{101}$ mode through a microstrip line and used a silver-coated slot pair to produce a lower-band null while shifting the higher-order $TM_{111}$ mode downward so that it forms a non-radiating upper-band null, yielding a structurally simple filtering response without additional circuitry. Ref. [25] merged the $TE_{12}$ and $TE_{1+\delta ,2}$ modes through a slot feed and used four metal cylinders to introduce a lower-edge null while the DP itself provided an upper-edge null, reshaping the dielectric from square to rectangular to suppress higher-order harmonics, and achieved high realized gain together with wide harmonic suppression. Ref. [27] generated three resonant modes, a first $TE_{11}$ from a centrally raised small DP plus a second $TE_{11}$ and a $TE_{12}$ from the main patch, and used parallel ground slots and a non-radiative dielectric mode to produce paired radiation nulls, while symmetric metal vias shifted the radiative and non-radiative $TE_{12}$ modes downward, resulting in an 8.2% bandwidth (3.75–4.07 GHz) and an average gain of 7.7 dBi. Ref. [26] proposed a dual-mode DP combining a high-permittivity ($\varepsilon_{r}=90$ ) dielectric with a low-permittivity substrate, with $TM_{11}$ degenerate modes derived analytically through the mixed magnetic-wall method and image theory, and demonstrated single-ended and differential bandpass filters with a low profile and high unloaded $Q_{u}$. Collectively, these works demonstrate that filtering response in DPAs is principally obtained through the merging of multiple dielectric modes combined with the deliberate introduction of radiation nulls, either intrinsically via non-radiative modes or extrinsically via etched slots, metal cylinders, vias, or geometric reshaping, with the trade-off between structural complexity, bandwidth, selectivity, and gain guiding the choice of technique.

MIMO configurations of DPAs have been pursued through multi-element arrangements that exploit the multi-mode nature of the DP to enhance port-to-port isolation without sacrificing bandwidth or radiation stability. Ref. [28] excited the TM_10_ and antiphase TM_20_ modes simultaneously in a slotted DPA, expanded the bandwidth by merging these modes through air tunnels, and neutralized the inductive inter-element coupling through capacitive coupling introduced by metal strips on the non-radiating edges, achieving a measured bandwidth of 17.9% (4.93–5.9 GHz) with port-to-port isolation exceeding 21.0 dB with a stable radiation pattern. Ref. [29] proposed a self-decoupling technique in which the attenuation constant of the fundamental TM_01_ mode outside the dielectric patch is controlled through the structural parameters of the resonator itself, so that higher attenuation yields stronger broadband isolation and pattern stability without any additional decoupling components. Ref. [30] exploited four resonant modes of the DP to form two orthogonally polarized wide operating bands, the latter excited differentially for high isolation, achieving bandwidths of 29.5% (4.71–6.1 GHz) and 18.9% (4.81–5.72 GHz) with isolation better than 48 dB across the band. Ref. [31] stacked two identical ceramic plates to generate highly pure adjacent modes with symmetrical field distributions and negligible spurious radiation, realizing a 42% bandwidth (3.3–5.15 GHz) with high isolation across the operating band for 5G/B5G base-station applications. Ref. [32] presented a ±45° dual-polarized heterogeneous stacked antenna composed of a driven metallic patch and a parasitic DP coupled through four off-center supporting dielectric blocks, where the low-frequency mode is generated by the metallic patch and the high-frequency mode by the DP, achieving an efficiency greater than 90% across a 14.1% bandwidth (3.3–3.8 GHz) with gain and half-power-beamwidth variations below 0.3 dBi and 5°, respectively. Collectively, these works demonstrate that MIMO performance in DPAs is principally governed by the exploitation of the multi-mode and field-distribution features of the DP, with the isolation mechanism shifting from external decoupling structures [28], through intrinsic parameter-driven field attenuation [29] and differential and orthogonal mode excitation [30,31], to hybrid metallic–dielectric stacking [32], each approach trading off structural complexity, bandwidth, isolation level, and radiation stability in a different manner.

Circular polarization (CP) in DPAs has been achieved primarily by exploiting degenerate or near-degenerate mode pairs inside the DP, which can be split through geometric perturbations to produce the two orthogonal components required for CP radiation. Ref. [33] selected two pairs of degenerate modes, TM_101_/TM_011_ and TM_121_/TM_211_, and introduced four air holes in the dielectric patch whose positions and sizes were tuned to bring both pairs into close spectral proximity, merging them to broaden the axial-ratio (AR) bandwidth and achieving a measured 3 dB AR bandwidth of 9.7% and a peak gain of 7.66 dBic at the X-band. Ref. [34] fused a square DP which has high permittivity ($\varepsilon_{r}=72$) with the substrate to form a resonator whose TM_101_/TM_011_ degenerate modes are split by perturbations on the DP and excited directly by a microstrip line, and demonstrated a 2×2 array fed by a dual Marchand balun with an impedance bandwidth of 380 MHz (5.18–5.56 GHz), a 3 dB AR bandwidth of 90 MHz (5.32–5.41 GHz), and a measured gain up to 11.77 dBic with −20 dB cross-polarization at boresight. Ref. [35] excited multiple modes of the feeding structure inside an air cavity together with two coupled DPs (permittivity of ($\varepsilon_{r}=21.6$ and 12) to widen the impedance bandwidth to 48.3% (4.63–7.58 GHz) and the AR bandwidth to 35.3% (5.15–7.36 GHz), with the upper DP acting as a director to raise the peak gain to 8.47 dBic at a self-packaged profile of $0.13\lambda_{0}$. Ref. [36] stacked two dielectric resonator layers with high and low permittivities (permittivities of $\varepsilon_{r}=45$ and 2.2) to merge two similar modes with adjacent resonant frequencies and used a single-feed coupling cross-slot to obtain CP operation, achieving an AR bandwidth of more than 20% and demonstrating the design concept at two frequency bands with opposite CP senses. Collectively, these works demonstrate that CP operation in DPAs is principally realized by splitting degenerate mode pairs through geometric perturbations [34] and by merging multiple degenerate or near-degenerate modes through air holes, stacked patches, or coupled resonators [33,35,36], with the trade-off between AR bandwidth, profile height, gain, and structural complexity determining the choice of technique for a given application.

The reported DPAs are predominantly implemented at sub-10 GHz frequencies, where the maturity of low-loss high-permittivity ceramic materials and the maturity of PCB-based multilayer fabrication have enabled the diverse mode-merging, null-engineering, isolation, and CP techniques summarized above. At mmWave frequencies, however, the existing DPA literature has largely focused on single-band operation, primarily targeting the 26–28 GHz 5G spectrum, while dual-band or multiband mmWave DPAs, which are required to support simultaneous multi-standard operation and Integrated Sensing and Communication (ISAC), remain comparatively unexplored. Bridging this gap between the design-rich sub-10 GHz and the mmWave band is the principal motivation of the present work.

The mmWave DPA literature is comparatively recent and has been shaped primarily by the 26–28 GHz 5G spectrum, with most reported designs prioritizing gain and bandwidth enhancement over compact, single-element, multiband functionality. Ref. [37] presented a 1×4 circular-shaped DP array fed by a Wilkinson power divider at 28 GHz, employing a compact uniplanar electromagnetic-bandgap ground structure and a dielectric superstrate layer for further gain improvement, and achieving an impedance bandwidth from 27 GHz to beyond 32 GHz with a total realized gain greater than 16 dBi; the trade-off is a multilayer assembly with array feeding and external superstrate loading. Ref. [38] proposed a 2×2 aperture-coupled square DP array at 28 GHz in which a holey dielectric superstrate with identical circular drilled holes simultaneously enhances the gain, improves the bandwidth, and reduces the sidelobe level, delivering a 15.35% impedance bandwidth (26.5–30.8 GHz), a flat measured gain of about 16 dBi, and a simulated radiation efficiency of 92%, similarly at the cost of an external superstrate and a 2×2 array. Ref. [39] used a highly reflective frequency-selective surface (FSS) superstrate to enhance the gain of a single aperture-coupled dense DP by approximately 11 dB, reaching about 17.78 dBi at 28 GHz with 9% bandwidth and 90% efficiency, and extended the bandwidth to 15.54% by printing the FSS unit cell on both sides of the superstrate to introduce a positive phase gradient, but at the expense of an additional multilayer FSS superstrate and a non-planar assembly. Ref. [40] introduced a compact mmWave dual-beam filtering antenna based on a DP resonator, in which coupling between the TE_10_ mode of a substrate-integrated cavity-backed double-slot and the TM_2*δ*_ mode of the dielectric strip forms the dual-beam band, while non-metallized vias in the high-field regions of the harmonic mode and a short-circuit coupling line provide out-of-band suppression and an upper-edge radiation null, achieving 11.4% fractional bandwidth at 26 GHz, 5.7 dBi peak gain, and a single radiation null at 28.6 GHz; this design operates as a single element but is restricted to a single band with 80% efficiency. Collectively, these works demonstrate that mmWave DPA performance to date has been achieved primarily through array configurations and external superstrate loading [37,38,39], which deliver high gain and moderate bandwidth but compromise the compact, single-element form factor inherent to the DPA architecture, while the single-element mmWave designs that exist [40] remain single-band and exhibit limited efficiency.

This paper proposes a compact tri-band DPA that simultaneously operates at 22, 30, and 36 GHz by exciting the fundamental ($TM_{11}$) and higher-order ($TM_{31}$, $TM_{51}$) modes of a single high-permittivity DP. Unlike all previously reported mmWave DPAs, the proposed design achieves multiband functionality from a single radiating element without resorting to array configurations, superstrate layers, or multilayer stacking. Full-wave simulations in CST Microwave Studio, validated through fabrication and experimental measurements of a prototype, demonstrate impedance bandwidths of 4.57%, 4.24%, and 2.75% at the three operating bands, respectively, while maintaining an ultra-low profile of only $0.086\lambda_{0}$ and radiation efficiencies of 93%, 95%, and 95% across all three bands.

## 2. Antenna Design and Configuration

**Figure 1:**
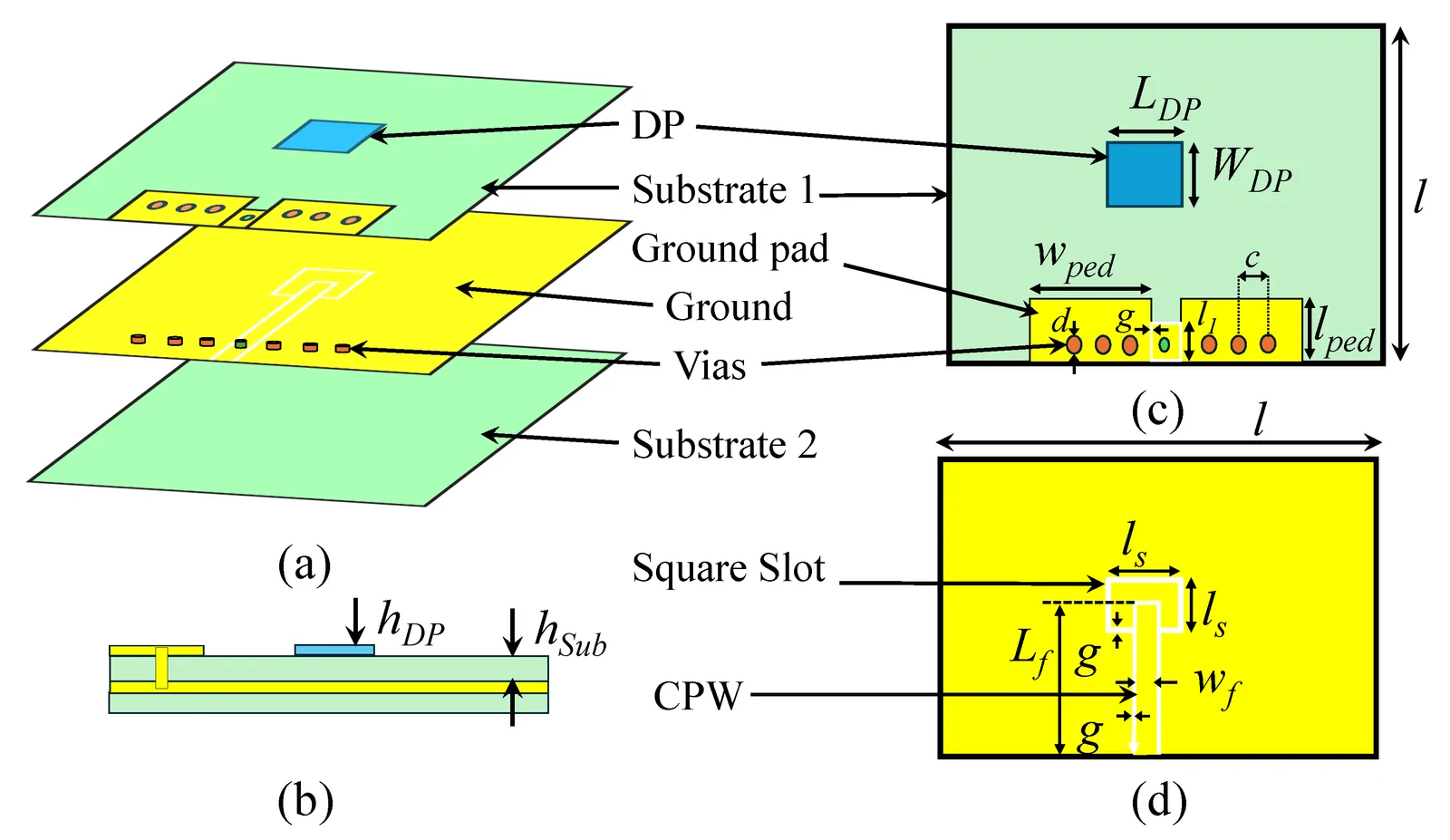
Geometry of the proposed tri-band dielectric patch antenna. (**a**) 3D view. (**b**) Side view. (**c**) Top view. (**d**) Ground plane.

The proposed configuration is illustrated in Figure 1. The antenna features a DPA constructed from a high-permittivity ceramic material with a relative permittivity (dielectric constant) of $\varepsilon_{rd}=82$ and a loss tangent of $\tan\delta =4.4\times 10^{-3}$. The DP has a thickness of $h_{DP}=0.15$ mm and dimensions of $L_{DP}=W_{DP}=4.5$ mm. The patch is mounted on the top surface of a Rogers RO4003C substrate with a relative permittivity of $\varepsilon_{rs}=3.55$, a loss tangent $\tan\delta =2.7\times 10^{-3}$, and a thickness $h_{Sub}=0.508$ mm.

**Figure 2:**
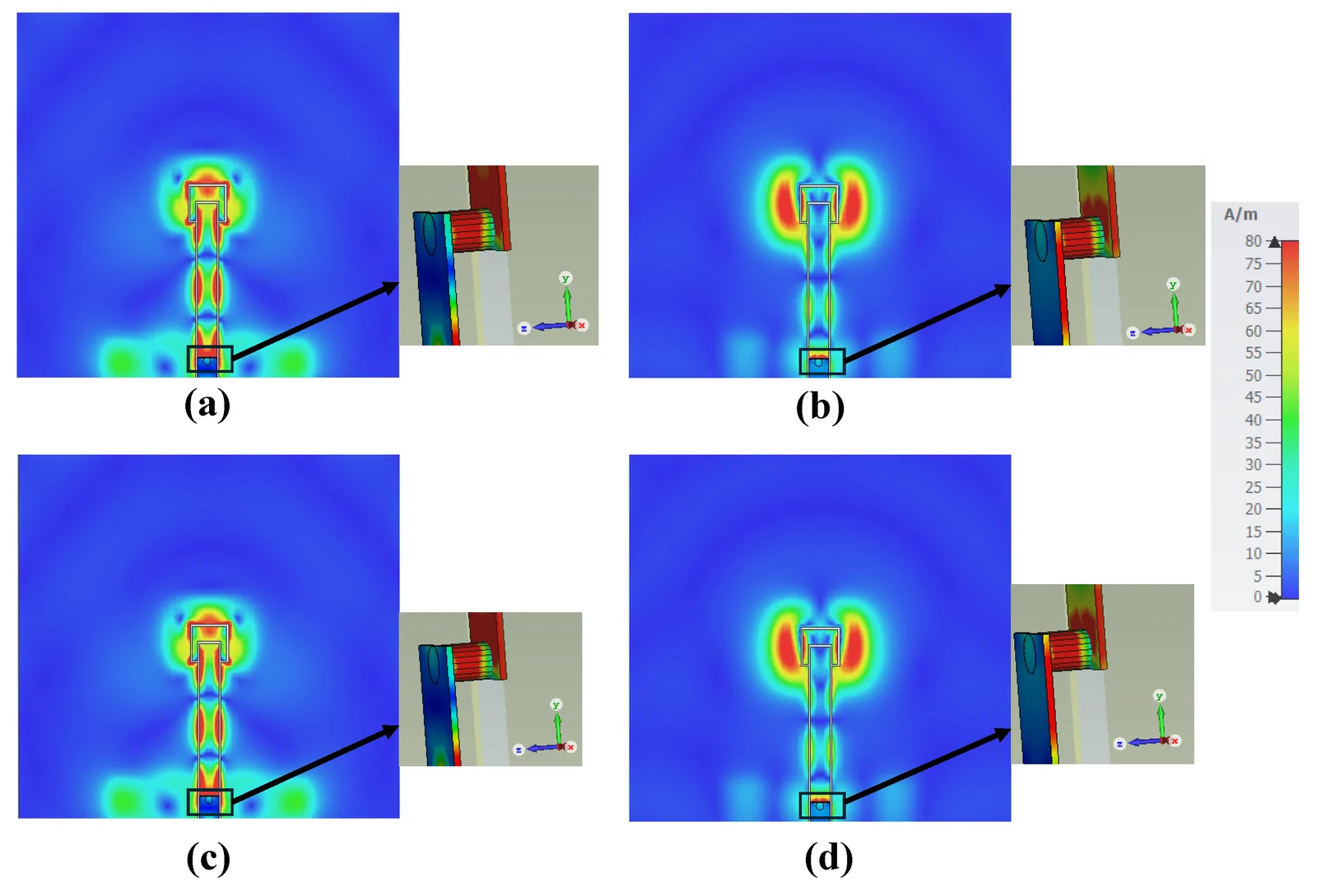
Simulated surface current distributions on the ground plane and vias at 30 GHz for different phase angles: (**a**) $\phi =0^{\circ }$, (**b**) $\phi =90^{\circ }$, (**c**) $\phi =180^{\circ }$, and (**d**) $\phi =270^{\circ }$. The side views illustrate the current flow through the vias of the CPW feed.

The bottom surface of the upper substrate (Substrate 1) serves as the ground plane, which incorporates an etched square slot that functions as an aperture-coupling feed mechanism. The 50-Ω CPW transmission line is fabricated on the top layer of the substrate and is connected to the ground plane via plated-through vias. The electromagnetic field generated by the CPW couples through the square aperture in the ground plane to excite the resonant modes of the DP, as illustrated in Figure 2. Because the primary CPW ground plane is embedded between the two substrate layers, small ground pads are utilized on the top layer, connected to the internal ground via metallic vias. This structure serves exclusively to connect the external RF connector to the buried ground plane, completing the electrical circuit and ensuring a continuous ground return path.

A second Rogers RO4003C substrate layer, identical in material properties and thickness, is stacked beneath the ground plane to complete the configuration. The metallization between the two RO4003C substrates serves as the ground plane. Substrate 2 provides lower half-space dielectric loading for symmetric permittivity, which stabilizes feed impedance. It functions as an electromagnetic buffer to ensure the dielectric patch is excited exclusively through the aperture by preventing parasitic coupling, while also providing structural support for standard PCB fabrication.

The DP was custom-procured from a commercial RF component provider to meet the target relative permittivity and specified physical dimensions. To ensure high precision during the physical assembly, a silkscreen outline was printed onto the top substrate layer during the standard PCB manufacturing process. This outline served as an exact alignment guide for the DP, which was then rigidly secured to the substrate using a thin layer of adhesive glue. Due to its minimal thickness, this bonding layer exerts a negligible effect on the antenna’s overall electromagnetic behavior. The optimized antenna parameters are summarized in Table 1.

## 3. Operating Mechanism and Mode Analysis

The multiband operation of the proposed antenna is attributed to the selective excitation of higher-order transverse-magnetic (TM) modes in the DP. The symmetric CPW feed and centrally located square aperture produce an excitation field with odd symmetry about the patch center. Consequently, the feed preferentially couples to the observed odd-order modes, which are identified from the simulated electric-field distributions as ${\mathrm{TM}}_{11}$, ${\mathrm{TM}}_{31}$, and ${\mathrm{TM}}_{51}$ at approximately 22, 30, and 36 GHz, respectively. In contrast, even-order modes are not evident within the investigated frequency range, which is attributed to their weaker coupling overlap with the aperture-excitation field.

### 3.1. Theoretical Analysis

To provide physical insight into the DP resonator’s behavior, an analytical model based on cavity theory and an anisotropic effective media framework, building on prior works [18,41,42,43], is used to clarify the resonant modes.

### 3.2. Resonator Model

The DP resonator consists of a high-permittivity substrate ($\varepsilon_{rd}=82$, $\tan\delta =4.4\times 10^{-3}$, dimensions $L_{DP}\times W_{DP}\times h_{DP}$ ) placed on a low-permittivity RO4003 substrate with ($\varepsilon_{rs}=3.55$, $\tan\delta =2.7\times 10^{-3}$ and thickness of $h_{s}$). The bottom surface is backed by a metallic ground plane, treated as a perfect electric wall (PEW) to effectively halve the resonator height. The four lateral sidewalls parallel to the *z*-axis are approximated as perfect magnetic walls (PMWs). Electromagnetic wave propagation is assumed along the *z*-direction.

Resonant modes are classified as TM_*mns*_, where *m*, *n*, and *s* denote half-wavelength variations along the *x*-, *y*-, and *z*-axes, respectively. The TM modes, dominant in this configuration, are characterized by a dominant electric-field component along the *z*-direction.

### 3.3. Anisotropic Effective Permittivity

For thicknesses much smaller than the wavelength ($h_{DP}+h_{sub}\ll \lambda_{0}$), the stacked structure homogenizes into an anisotropic medium with a diagonal permittivity tensor [44]:

$$\varepsilon_{r}=\left[\begin{array}{ccc} \varepsilon_{rx} & 0 & 0\\ 0 & \varepsilon_{ry} & 0\\ 0 & 0 & \varepsilon_{rz} \end{array}\right].$$

where $\varepsilon_{rx}=\varepsilon_{ry}$ due to symmetry, and the tensor components are derived via weighted averages for tangential directions and series capacitance for the normal direction [41]:

(1) $$\varepsilon_{rx}=\varepsilon_{r(DP)}+\left(\varepsilon_{r(sub)}-\varepsilon_{r(DP)}\right)\times \left(\frac{h_{sub}}{h_{DP}+h_{sub}}\right),$$

(2) $$\varepsilon_{rz}={\left(\frac{1}{\varepsilon_{r(DP)}}-\frac{\varepsilon_{r(sub)}-\varepsilon_{r(DP)}}{\varepsilon_{r(DP)}\varepsilon_{r(sub)}}\times \left(\frac{h_{sub}}{h_{DP}+h_{sub}}\right)\right)}^{-1}.$$

For the given values ($\varepsilon_{r(DP)}=82$, $\varepsilon_{r(sub)}=3.55$, $h_{DP}=0.15$ mm, $h_{sub}=0.5$ mm), this yields $\varepsilon_{rx}\approx 21.65$ and $\varepsilon_{rz}\approx 4.56$, resulting in directional electromagnetic-field confinement as per [42,43,45].

### 3.4. Resonant Frequency Derivation TMmn Mode

For ${\mathrm{TM}}_{mns}$ modes, the structure may be represented by an equivalent anisotropic resonator with [18]:

(3) $$\varepsilon_{r,\mathrm{eff}}=\frac{\left(\varepsilon_{rx}\times m+\varepsilon_{ry}\times n+\varepsilon_{rz}\times s\right)}{\left(m+n+s\right)}$$

(4) $$h_{\mathrm{eff}}=2\times \left(h_{DP}+h_{sub}\right)$$

Wavenumbers satisfy

(5) $$k_{x}=\frac{m\pi }{L_{DP}},\;k_{y}=\frac{n\pi }{W_{DP}},\;k_{z}=\frac{2\arctan\left(\frac{\varepsilon_{r,\mathrm{eff}}\alpha_{z}}{k_{z}}\right)}{h_{\mathrm{eff}}}$$

(6) $$k_{z}^{2}+\alpha_{z}^{2}=\left(\varepsilon_{r,\mathrm{eff}}-1\right)\times k_{0}^{2},\;k_{0}=\frac{2\pi f}{c}.$$

The resonant frequency of a given mode is expressed as

(7) $$f=\frac{c}{2\pi \sqrt{\varepsilon_{r,\mathrm{eff}}}}\sqrt{k_{x}^{2}+k_{y}^{2}+k_{z}^{2}}.$$

where $k_{x}$, $k_{y}$, and $k_{z}$ represent the wavenumbers in the *x*-, *y*-, and *z*-directions, respectively; $\alpha_{z}$ denotes the dissipation mode wavenumber along the *z*-axis; and $k_{0}$ is the free-space wavenumber at the operating frequency.

Using the proposed antenna dimensions, the calculated resonant frequencies associated with the three excited modes are 21.8, 29.7, and 35.4 GHz. These values are close to the simulated resonances at 21.5, 30, and 36 GHz, as well as the measured resonances near 22, 30, and 36 GHz, respectively. This semi-analytical model assumes magnetic sidewalls and an electric wall at the ground plane, as in the equivalent cavity model of [18]. Accordingly, it provides an approximate modal-frequency estimate, with the final modal identification verified using full-wave simulations.

**Figure 3:**
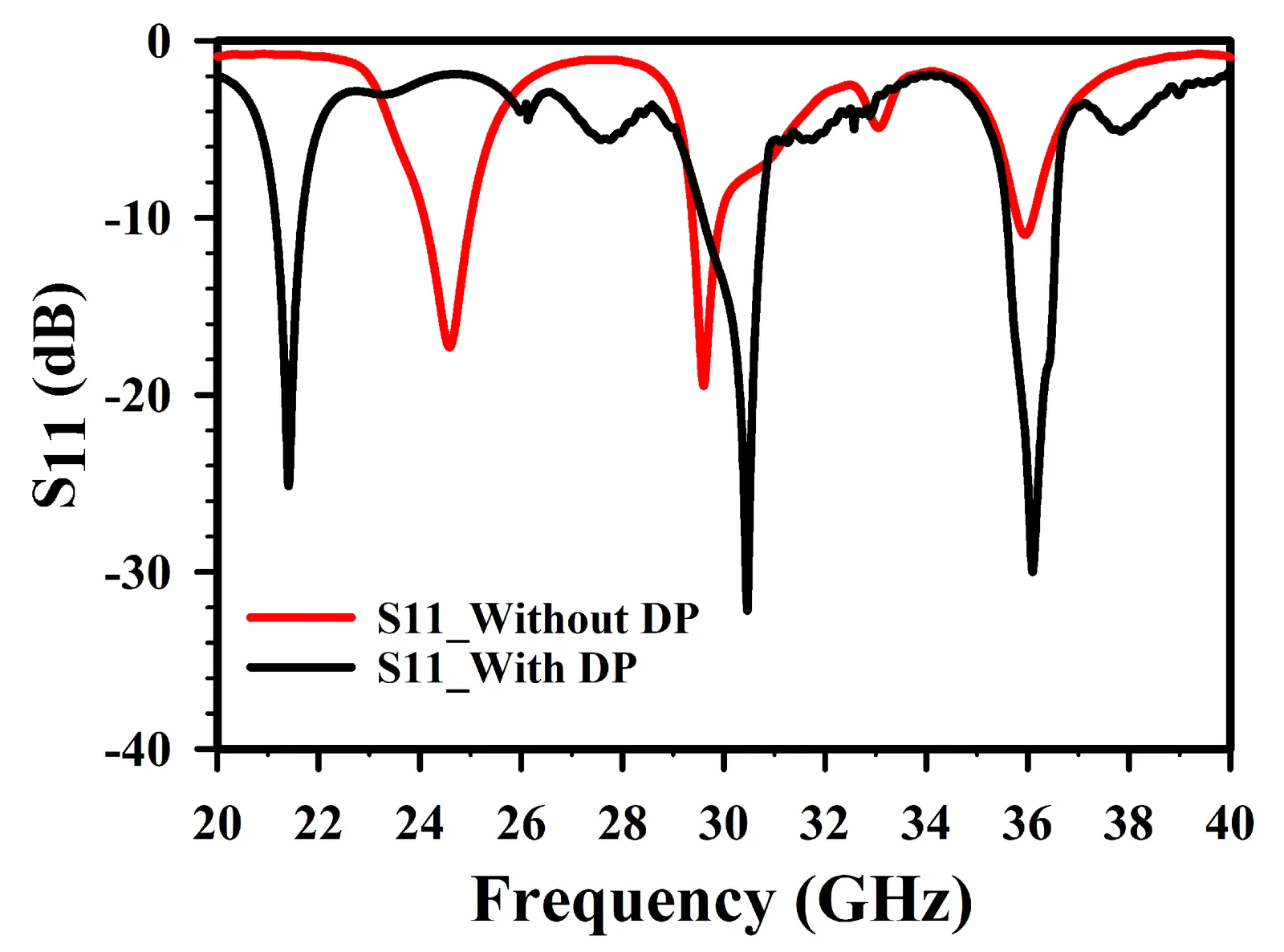
Simulated reflection coefficient of the complete antenna (with DP) and of the feeding structure alone (without DP).

**Figure 4:**
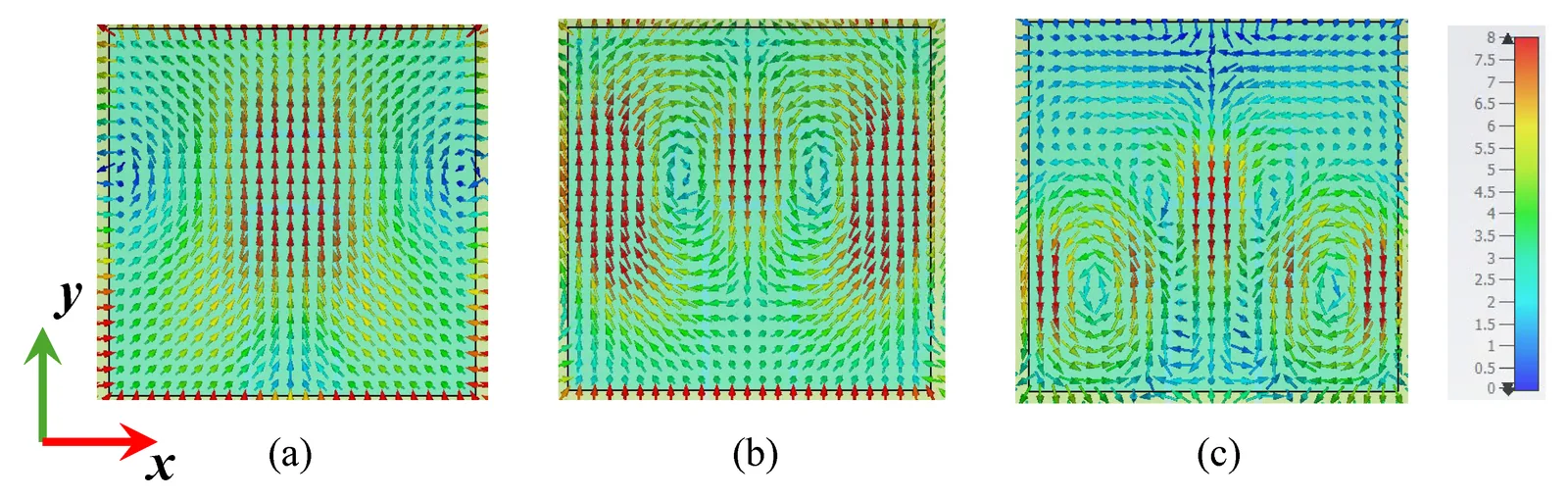
Simulated electric-field vectors on the field magnitude |E| on a cut inside the dielectric patch at the resonant frequencies (**a**) 21.5 GHz (${\mathrm{TM}}_{11}$), (**b**) 30 GHz (${\mathrm{TM}}_{31}$), and (**c**) 36 GHz (${\mathrm{TM}}_{51}$).

**Figure 5:**
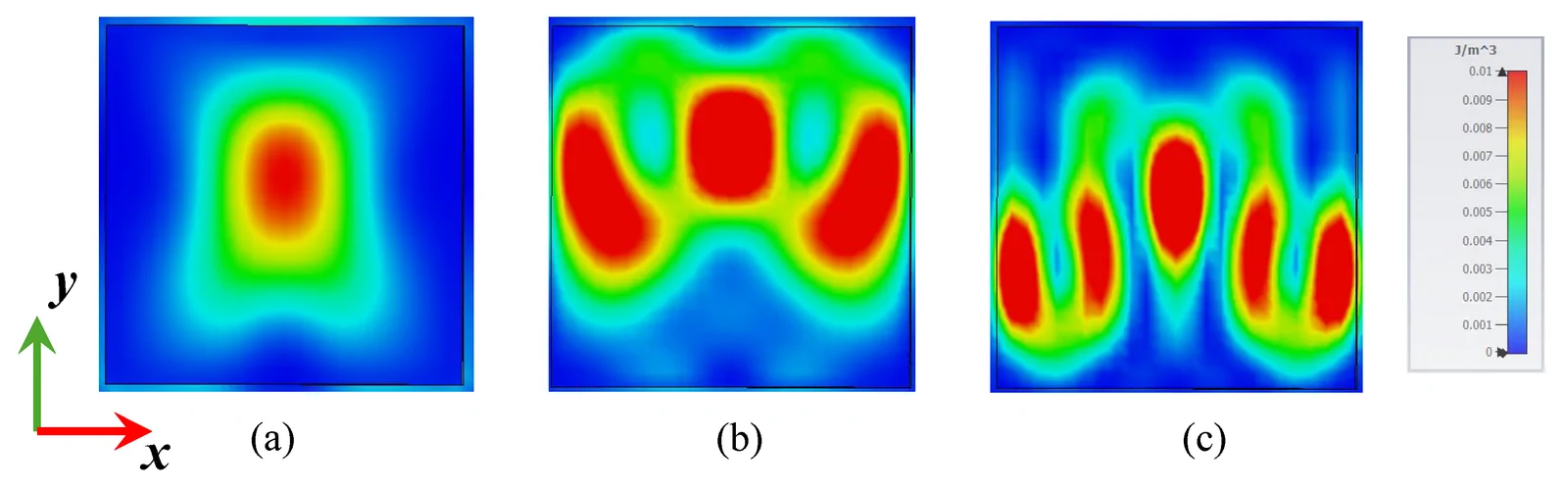
Simulated electric-energy density inside the dielectric patch at (**a**) 21.5 GHz (${\mathrm{TM}}_{11}$), (**b**) 30 GHz (${\mathrm{TM}}_{31}$), and (**c**) 36 GHz (${\mathrm{TM}}_{51}$).

To distinguish the dielectric patch resonances from those of the feeding network, $S_{11}$ was computed with and without the DP (Figure 3). The CPW and square aperture alone remain resonant, as expected for a slot-coupled feed, with matches near 24.5 GHz and in the vicinity of 30 and 36 GHz, but with no match near 22 GHz. Restoring the DP creates the lowest operating band and substantially deepens (and slightly retunes) the upper two matches. At the three matched frequencies of the complete antenna, the in-plane field distributions and the electric-energy density inside the DP (Figure 4 and Figure 5) exhibit one, three, and five variations along *x*, consistent with ${\mathrm{TM}}_{11}$, ${\mathrm{TM}}_{31}$, and ${\mathrm{TM}}_{51}$. The feed therefore provides coupling and can exhibit its own slot resonances, while the DP modes govern the tri-band response.

## 4. Parametric Study

**Figure 6:**
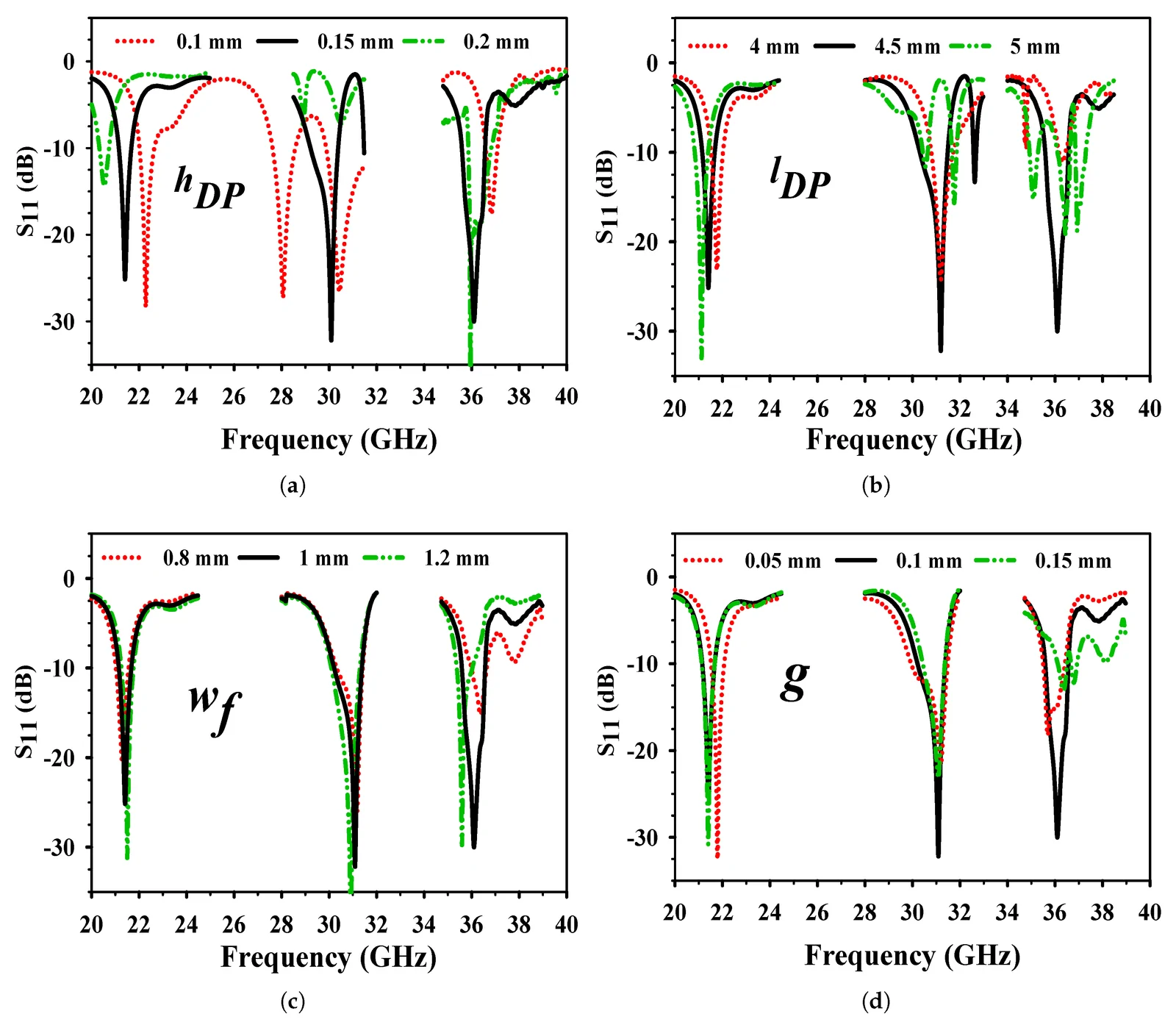
Simulated $S_{11}$ variation for different parameter values. (**a**) Varying DP height $h_{DP}$. (**b**) Varying DP length $l_{DP}$. (**c**) Varying CPW feed width $w_{f}$. (**d**) Varying gap width *g*. In each case, other parameters are fixed at the optimized values.

To provide physical insight into the operating mechanism of the proposed multiband antenna and to verify the robustness of the design, a systematic parametric study of the key geometrical variables is presented in Figure 6. In each case, a single parameter is swept while all remaining dimensions are held at their nominal optimized values, thereby isolating the individual contribution of each variable to the reflection-coefficient ($S_{11}$) response across the three operating bands (centered at approximately 22 GHz, 30 GHz, and 36 GHz). This approach not only clarifies the underlying electromagnetic behavior but also establishes the antenna’s sensitivity to each degree of freedom.

### 4.1. Effect of the Dielectric Patch Height, ($h_{DP}$)

As illustrated in Figure 6a, the DP height $h_{DP}$ predominantly governs the depth of the impedance match across all three bands, whereas its influence on the resonant frequencies remains comparatively marginal. Specifically, as $h_{DP}$ is increased from 0.1 mm through 0.15 mm to 0.2 mm, the resonant dips deepen appreciably, with the matching level improving towards −30 dB at the optimum value of 0.15 mm. This behavior may be attributed to the fact that variations in $h_{DP}$ alter the effective coupling volume of the resonator and, in turn, modify the magnitude of the reactive energy exchanged between the feed and the radiating structure. Consequently, $h_{DP}$ functions primarily as an impedance-matching control rather than a frequency-tuning parameter, since the electrical length that dictates resonance is only weakly perturbed. However, it is worth noting that an excessive increase in $h_{DP}$ tends to degrade the match in the higher band, thereby indicating the existence of an optimal trade-off value.

### 4.2. Effect of the Patch Length, ($L_{DP}$)

In contrast to $h_{DP}$, the DP length $L_{DP}$ exerts a critical influence on the resonant frequencies, as demonstrated in Figure 6b. As $L_{DP}$ is increased from 4 mm to 4.5 mm and subsequently to 5 mm, a systematic and predictable downward shift towards lower frequencies is observed uniformly across all three bands. This similar trend is a direct consequence of the enlarged electrical length of the resonator: since the resonant frequency is inversely proportional to the effective current path, a longer patch supports resonance at a correspondingly lower frequency. Importantly, the near-uniform shift in all bands suggests that the multiple resonances originate from higher-order modes of a common radiating element, rather than from independent structures. Accordingly, $L_{DP}$ represents the principal frequency-tuning parameter of the design, enabling the operating bands to be relocated.

### 4.3. Effect of the CPW Feed Width, ($w_{f}$)

The influence of the CPW feed parameters is examined in Figure 6c,d. As shown in Figure 6c, varying the signal-strip width $w_{f}$ from 0.8 mm to 1.2 mm produces only negligible displacement of the resonant frequencies; the band centers remain fixed. Nevertheless, $w_{f}$ has a noticeable effect on the quality of the impedance match, particularly within the upper band, where the nominal value of 1 mm yields the deepest resonance (approaching −30 dB at ∼36 GHz). Therefore, the strip width, in conjunction with the slot gap, determines the characteristic impedance of the feed line; consequently, $w_{f}$ serves as a fine-tuning mechanism for achieving the 50Ω input match rather than as a frequency- selective parameter.

### 4.4. Effect of Aperture and the CPW Gap, (*g*)

A closely related behavior is observed for aperture and the CPW slot gap *g* in Figure 6d. As *g* is swept from 0.05 mm through 0.1 mm to 0.15 mm, the three resonant dips (at ∼22 GHz, ∼30 GHz, and ∼36 GHz) remain virtually stationary in frequency, thereby confirming that the gap does not participate directly in setting the resonant condition. Instead, *g* modulates the reflection level by adjusting the characteristic impedance and the field confinement of the coplanar line. Hence, together with $w_{f}$, the gap *g* provides an additional and largely orthogonal degree of freedom for optimizing the 50Ω transition.

## 5. Design Procedure

To achieve predictable tri-band operation, the proposed antenna was synthesized through the following systematic procedure:

1. **Fundamental-mode calculation.** The initial resonant frequency of the ${\mathrm{TM}}_{11}$ mode is calculated using the established analytical equations. This theoretical baseline relies on the physical dimensions ($L_{\mathrm{DP}}$, $W_{\mathrm{DP}}$, $h_{\mathrm{DP}}$), the relative permittivity of the available commercial DP material, and the substrate parameters.
2. **Higher-order mode frequencies.** With the same geometry, the ${\mathrm{TM}}_{31}$ and ${\mathrm{TM}}_{51}$ frequencies are calculated from the same modal formula. This step confirms whether the intrinsic multiple-resonance profile of the selected DP parameters aligns with the targeted 30 GHz and 36 GHz millimeter-wave bands. If it does not, the DP parameters are adjusted and steps 1–2 are repeated until the three predicted frequencies coincide with the target bands.
3. **Coupling and feed.** A square aperture is introduced in the ground plane to provide magnetic coupling from the feed into the DP. A coplanar waveguide (CPW) is selected as the feeding method in order to drive the aperture efficiently while retaining a strictly planar, easily integrable, and adjustable layout.
4. **Optimization of the slot and CPW.** The aperture coupling and the CPW strip parameters ($L_{f}$, $l_{s}$, *g*, $W_{f}$) are then optimized. These parameters primarily control coupling strength and impedance matching rather than the modal frequencies, which remain governed by the DP. The optimization is continued until deep, simultaneous matches are obtained at the three predicted resonances.

## 6. Results and Discussion

**Figure 7:**
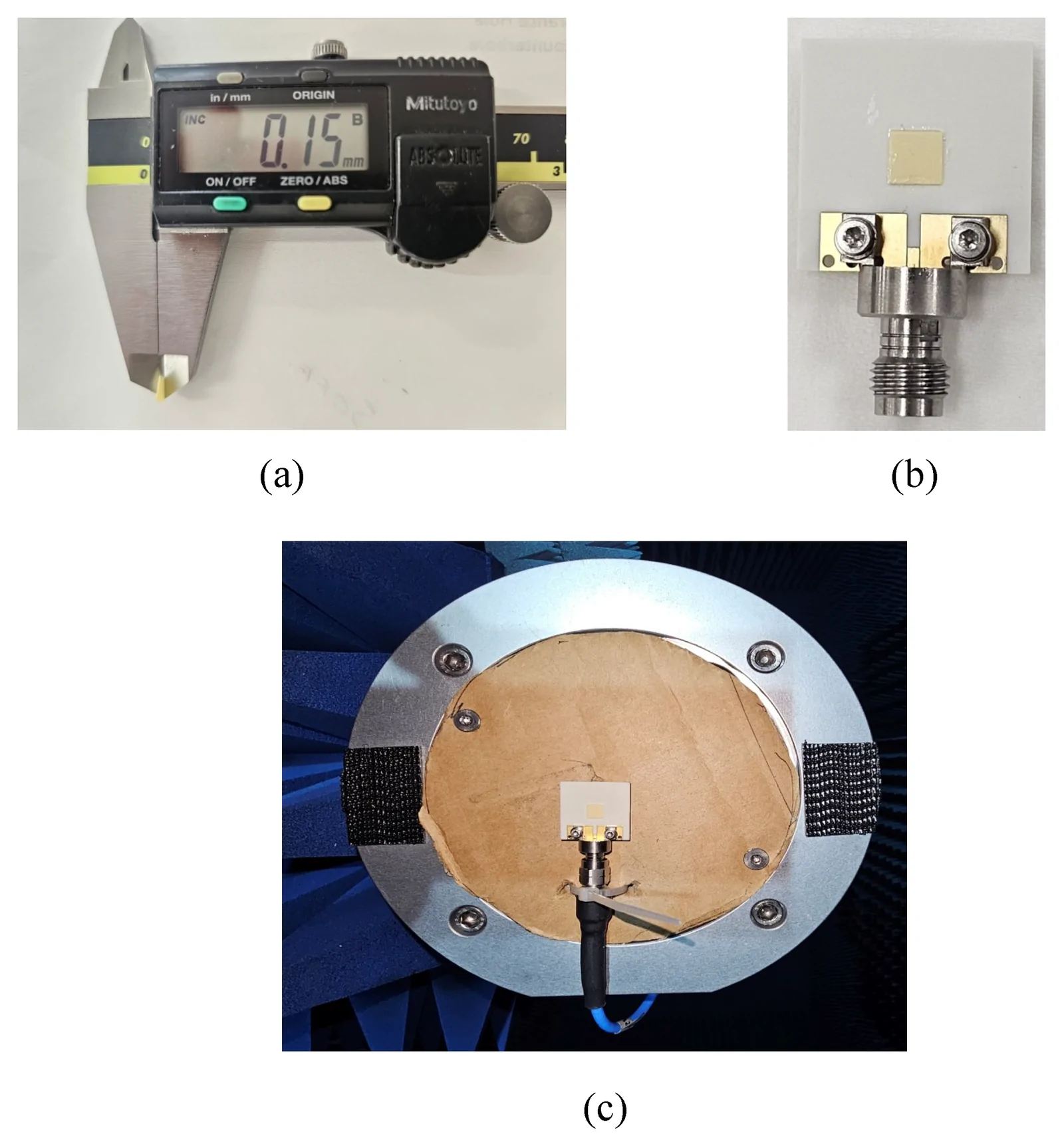
Photographs of the fabricated proposed multiband DPA and its measurement setup: (**a**) DP element during thickness measurement, (**b**) fabricated and assembled antenna prototype, and (**c**) AUT inside the anechoic chamber.

The proposed antenna was fabricated, assembled with a 2.4 mm connector and tested, as illustrated in Figure 7. The reflection coefficient was measured using a calibrated vector network analyzer (VNA). The far-field radiation patterns and realized gain were measured in an anechoic chamber using a standard gain-comparison method.

**Figure 8:**
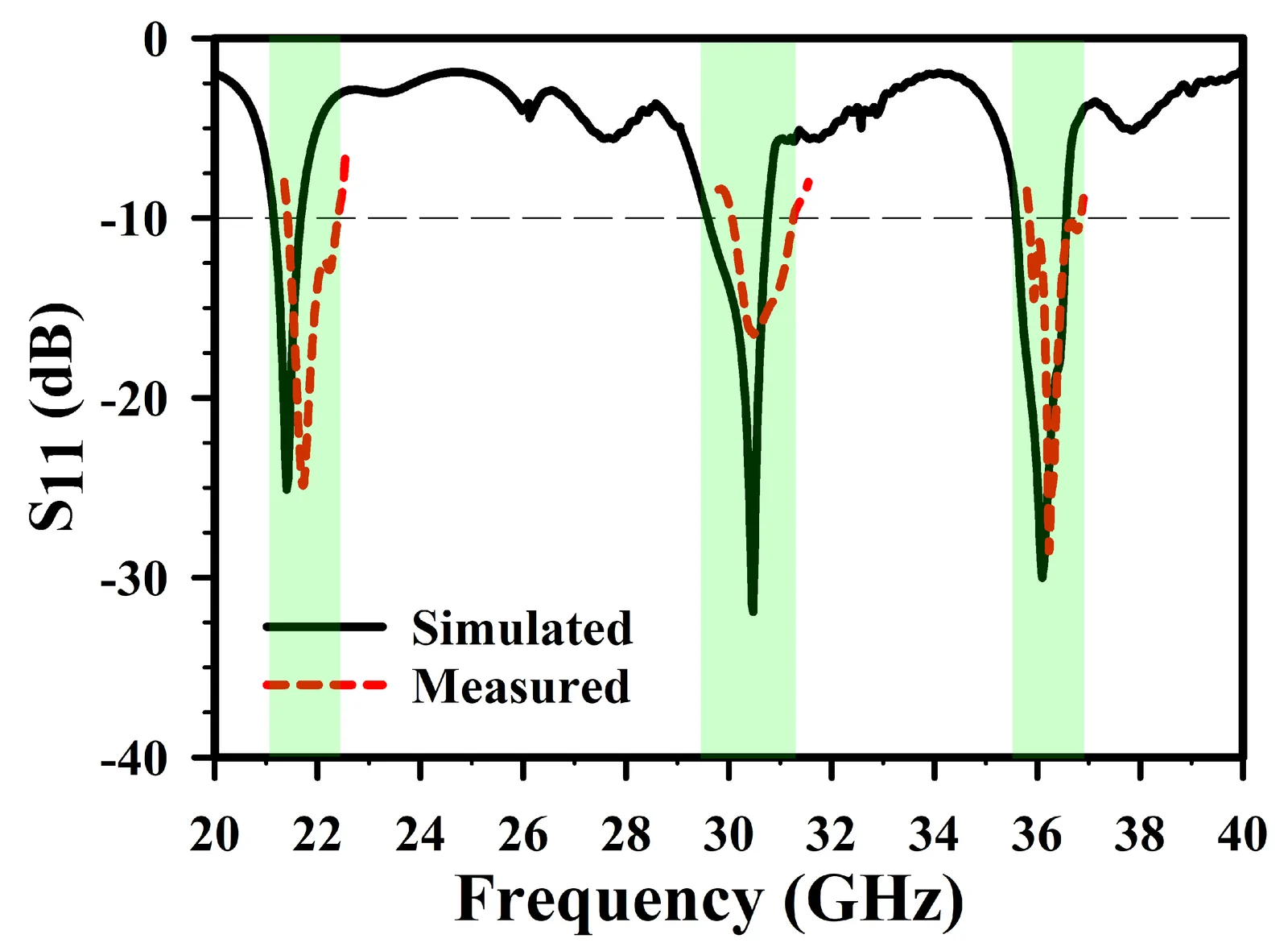
Simulated and measured reflection coefficient ($S_{11}$) of the proposed antenna.

**Figure 9:**
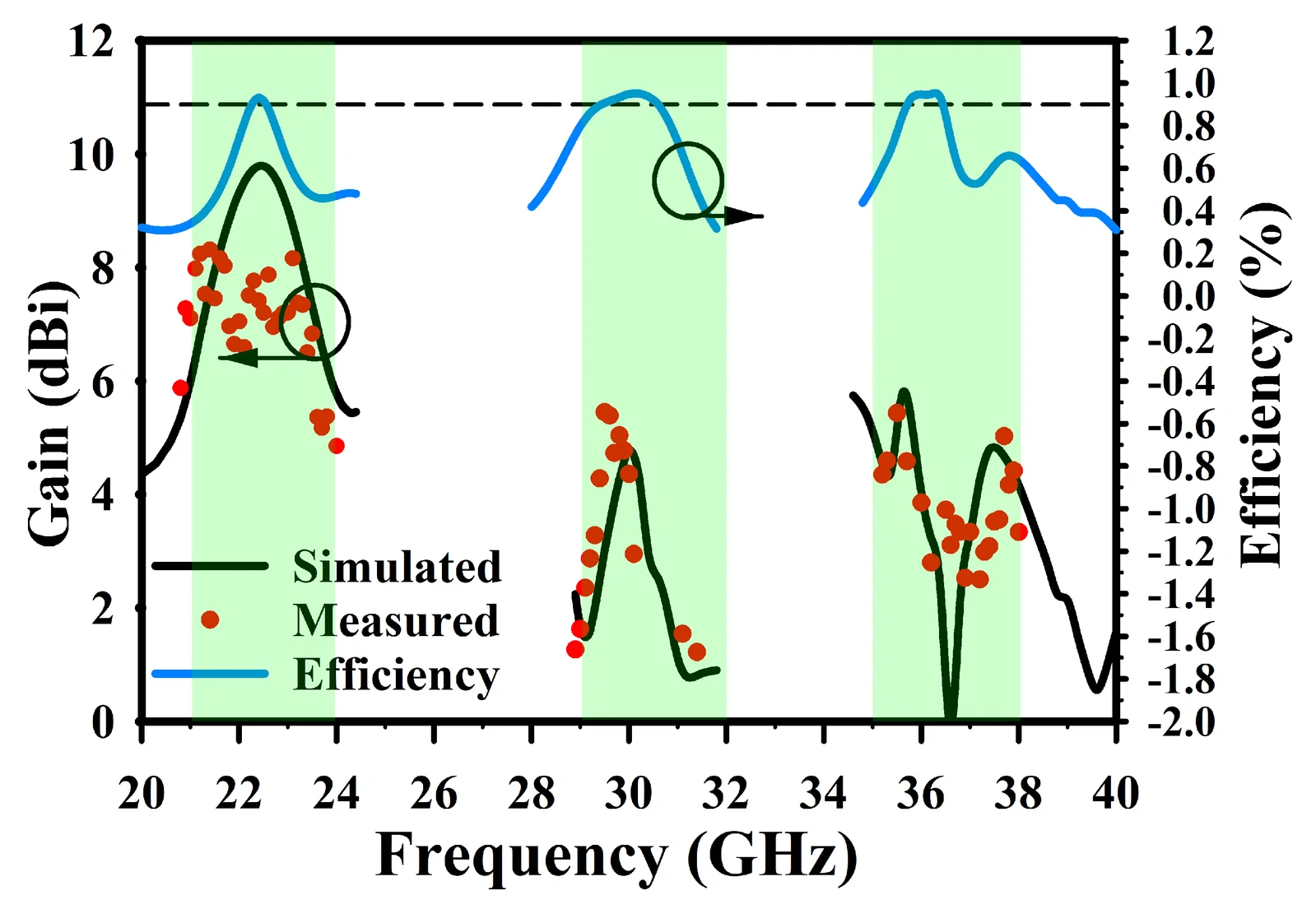
Simulated and measured realized gain and simulated radiation efficiency.

Figure 8 compares the simulated and measured reflection coefficients ($|S_{11}|$). Figure 9 compares the simulated and measured realized gains and presents the simulated radiation efficiency.

The measured and simulated $|S_{11}|$ results are in good agreement. Three distinct impedance-matched bands are clearly identified, centered at approximately 22, 30, and 36 GHz with measured impedance bandwidths of 4.57% (21.4–22.4 GHz), 4.24% (30–31.3 GHz), and 2.75% (35.8–36.8 GHz), respectively, compared to simulated bandwidths of 2.80% (21.1–21.7 GHz), 4% (29.6–30.8 GHz), and 3% (35.5–36.6 GHz). Minor discrepancies between the simulated and measured $|S_{11}|$ traces are primarily attributed to fabrication tolerances, manual placement and alignment of the high-permittivity DP, and slight deviations in the actual material properties (especially the loss tangent of the ceramic) at mmWave frequencies.

Regarding the gain performance (Figure 9), the measured peak realized gains of approximately 8.3, 5.2, and 4.3 dBi were obtained at 22, 30, and 36 GHz, corresponding to the ${\mathrm{TM}}_{11}$, ${\mathrm{TM}}_{31}$, and ${\mathrm{TM}}_{51}$ modes, respectively. These results closely follow the simulated values of 9.5, 5.5 and 4.7 dBi, with only marginal deviations across the three operating bands. It is observed that the measured gain at the lowest band is lower than the idealized simulated values. This discrepancy is largely attributed to practical millimeter-wave measurement challenges, including insertion losses at the physical 2.4 mm connector-to-CPW transition, minor fabrication tolerances in the multilayer substrate stack-up, and slight alignment variations during the anechoic chamber measurement.

Furthermore, the simulated radiation efficiency remains above 90%, peaking at approximately 93%, 95%, and 95% at the three operating frequencies, respectively. The sustained high efficiency across the three well-separated frequency bands confirms the low-loss nature of the DPA and validates the effectiveness of the proposed design.

It should be noted that these simulated efficiency values represent an idealized upper bound. In the fabricated mmWave prototype, the practical radiation efficiency is expected to be slightly lower. This reduction is primarily attributed to unmodeled physical factors inherent to high-frequency fabrication, including copper surface roughness, the finite conductivity of the adhesive bonding layer used to secure the DP, and realistic material dispersion. The strong agreement between the simulated and measured peak realized gains assist with confidence in the highly efficient radiating characteristics of the proposed antenna.

**Figure 10:**
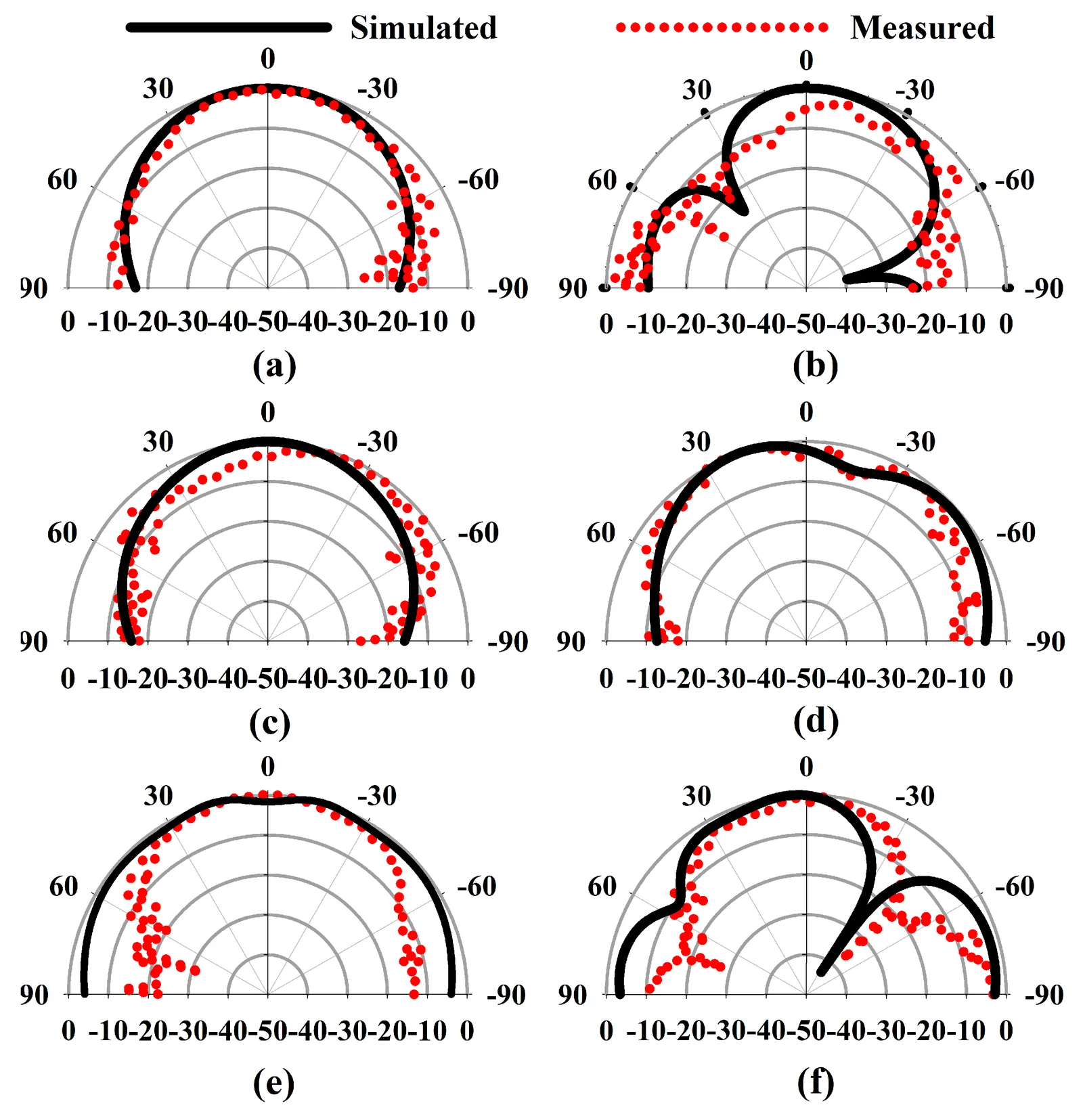
Simulated and measured normalized radiation patterns at 22 GHz for (**a**) $\phi =0^{\circ }$ and (**b**) $\phi =90^{\circ }$, at 30 GHz for (**c**) $\phi =0^{\circ }$ and (**d**) $\phi =90^{\circ }$, and at 36 GHz for (**e**) $\phi =0^{\circ }$ and (**f**) $\phi =90^{\circ }$.

The simulated and measured radiation patterns in Figure 10 show good agreement at the three representative operating frequencies. At 22 GHz, both $\phi =0^{\circ }$ and $\phi =90^{\circ }$ cuts exhibit symmetric patterns with maximum radiation near broadside. At 30 GHz and 36 GHz, the higher-order modes produce more pronounced split radiation patterns and broadside depressions. The close match between simulation and measurement validates the design and fabrication process, with minor discrepancies attributable to fabrication tolerances as well as misalignment in the far-field measurement setup.

The radiation patterns of these specific modes are highly suitable for their corresponding millimeter-wave applications. The fundamental ${\mathrm{TM}}_{11}$ mode at 22 GHz produces a broad, boresight-directed beam, which is ideal for direct point-to-point links such as fixed microwave backhaul. Conversely, the higher-order ${\mathrm{TM}}_{31}$ (30 GHz) and ${\mathrm{TM}}_{51}$ (36 GHz) bands—which serve Ka-band and 5G NR n257/n258/n260 applications—exhibit split, multilobe radiation patterns with off-boresight peak gains. Rather than being a drawback, these multidirectional beams provide valuable spatial diversity. In dense 5G and beyond-5G (B5G) indoor environments, signals suffer from severe line-of-sight blockage. Therefore, the wider angular coverage afforded by these split lobes is highly advantageous for indoor access points and IoT hubs communicating with randomly distributed devices.

Table 2 presents a performance comparison between the proposed antenna and recently reported mmWave multiband designs, categorized by antenna structure.

First, we detail a comparison to the broad literature of metallic-patch (MP) antennas [4,6,7,8,9,10,11,12,13]. For example, compared to the tri-band HMSIW-fed patch in [7], the proposed DPA features notable structural simplicity, generating three operating bands without relying on complex internal slits or intricate patch modifications. Because the primary radiating element is dielectric, the proposed DPA provides consistently high efficiencies across all three bands (≥93%) due to diminished conductor loss. In contrast, several miniaturized or multilayered MP designs in the comparison suffer from severe efficiency degradation, with reported values dropping to 30–50% [9] and 54–60% [13]. Furthermore, the proposed design is backed by a distinct theoretical framework governing the ${\mathrm{TM}}_{11}$, ${\mathrm{TM}}_{31}$, and ${\mathrm{TM}}_{51}$ modes, enabling systematic modification of the working frequencies based on selected geometric and material parameters. The slightly thicker profile ($0.086\lambda_{0}$) compared with the ultra-thin $0.03\lambda_{0}$ in [7] is acknowledged as a characteristic trade-off of the dielectric resonator topology.

Second, the comparison reveals a distinct gap in the existing dielectric antenna literature. Prior mmWave DPAs predominantly rely on multi-element arrays to achieve high absolute gain [37,38,39] or are strictly limited to single-band operation in single-element configurations [40]. The proposed antenna is the only single-element radiator achieving tri-band mmWave operation while simultaneously offering an exceptionally low dielectric profile. At $0.086\lambda_{0}$ (where $\lambda_{0}$ is the free-space wavelength at 22 GHz), it is notably thinner than the only other reported multiband dielectric antenna in this comparison, the DRA in [16], representing a profile reduction of approximately 47%.

Finally, while the proposed DPA exhibits varying peak gain across its three bands (8.0, 5.2, and 4.3 dBi), this mode-dependent variance is highly consistent with the behavior of other multi-resonant structures. Similar reductions or fluctuations in broadside gain at higher resonant frequencies have been widely reported in other multiband DRAs [16] and MPs [6,13], reflecting the inherent challenge of maintaining uniform aperture illumination and phase distribution across fundamentally different higher-order radiating modes. Ultimately, the proposed single-element design offers a practically favorable and highly efficient trade-off between physical compactness, multiband flexibility, and severe conductor-loss mitigation.

## 7. Conclusions

In this paper, a compact, tri-band dielectric patch antenna (DPA) fed by a coplanar waveguide (CPW) has been proposed and experimentally validated for 5G and beyond millimeter-wave (mmWave) applications. The design achieves simultaneous operation at three technologically significant frequency bands—22, 30, and 36 GHz—by predictably exciting the ${\mathrm{TM}}_{11}$, ${\mathrm{TM}}_{31}$, and ${\mathrm{TM}}_{51}$ modes of a single dielectric resonator, supported by a theoretical framework. These targeted bands explicitly address the spectral demands of modern wireless infrastructures, corresponding to fixed short-range backhaul links (22 GHz), 5G NR n257/n258 allocations (30 GHz), and an early 5G NR n260 spectrum integrated with radar and sensing applications (36 GHz). Experimental measurements demonstrate firm agreement with full-wave simulations, yielding fractional impedance bandwidths of 4.57%, 4.24%, and 2.75%, alongside measured peak realized gains of 8.3, 5.2, and 4.3 dBi, and exceptionally high simulated radiation efficiencies (≥93%) across the three respective bands. Overall, the proposed antenna effectively mitigates the trade-off between profile and performance, offering a highly favorable combination of compactness, application-specific tri-band flexibility, fabrication simplicity, and robust efficiency for next-generation mmWave wireless communication system front ends. 

## Figures and Tables

**Table 1:** Optimized dimensions of the proposed antenna.

Parameter	Value (mm)	Parameter	Value (mm)
*l*	20	$h_{\mathrm{Sub}}$	0.508
$L_{\mathrm{DP}}=W_{\mathrm{DP}}$	4.5	$h_{\mathrm{DP}}$	0.15
$L_{f}$	8.40	$l_{s}$	2
*g*	0.1	$W_{f}$	1
*d*	1	*c*	2
$W_{\mathrm{ped}}$	4.40	$l_{\mathrm{ped}}$	3

**Table 2:** Performance comparison of the proposed antenna with selected reported mmwave antenna designs.

Ref.	$f_{0}$ (GHz)	Gain (dBi)	Eff. (%)	Profile ($\lambda_{0}$)	Structure
[4]	25.5/38	7.4/7.9	–	–	MP
[6]	24.5/28.8/39	5.4/2.8/7.7	92/79/95	–	MP
[7]	28.6/34.1/39	7.6/8.7/8.45	87/90/92	0.03	MP
[8]	27/40	8/9.1	95	0.075	MP
[9]	28/38/61.5	1.4/3.1/4.5	50/30/45	–	MP
[10]	28/43/52/57	7.3 /7/7.2/8.0	86.5/87.5/89.2/90	0.48	MP
[11]	28/38/60	5.3/7.5/9.0	>85	–	MP
[12]	29/38/55	6.6/7.0/7.3	>85	–	MP
[13]	28/38/61	8.1/8.3/7.5	54/60/58	–	MP
[5]	24.1/29.9/36.2	5.5/5.8/9.7	–	–	SIW
[16]	17.5/23/28.5	7.3/6.8/5.8	90/87/84	0.16	DRA
[37]	28	16.3	71	–	DPA array
[38]	28	15.3	90	–	DPA array
[39]	28	15.5	92	–	DPA array
[40]	26	5.7	80	–	DPA
**This work**	**22/30/36**	**8.3/5.2/4.3**	**93/95/95**	**0.086**	**DPA**

MP: Microstrip Patch. DRA: Dielectric Resonator Antenna. DPA: Dielectric Patch Antenna. SIW: Substrate-Integrated Waveguide.

## Data Availability

Data are contained within the article.
